# Supplementary material for: Long Noncoding RNA AROD Inhibits Host Antiviral Innate Immunity via the miR-324-5p–CUEDC2 Axis
Source: Microbiol Spectr. 2023 Apr 10;11(3):e04206-22. doi: 10.1128/spectrum.04206-22 (PMC10269697; doi:10.1128/spectrum.04206-22)
Supplement: Supplemental file 1 — Fig. S1. Download spectrum.04206-22-s0001.pdf, PDF file, 0.05 MB [file spectrum.04206-22-s0001.pdf]

Supplemental Figure 1

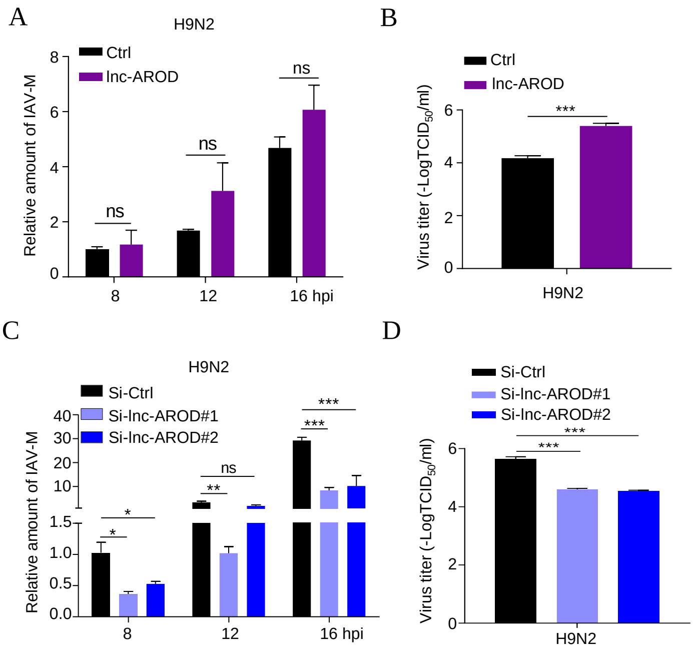

Supplemental Figure 1. Lnc-AROD promotes H9N2 viral replication. (A) The level of viral M mRNA expression was examined in lnc-AROD-overexpressing cells by qRT-PCR at the indicated time points. (B) The H9N2 viral titer in the supernatants were measured in lnc-AROD-overexpressing cells at 48 hpi. (C) The levels of viral M mRNA expression was examined in A549 cells transfected with lnc-AROD-specific siRNA by qRT-PCR at the indicated time points. (D). The H9N2 viral titer in supernatants were measured in A549 cells transfected with lnc-AROD-specific siRNA at 48 hpi. The data are expressed as the means of three independent experiments. *$P < 0.05$; **$P < 0.01$; ***$P < 0.001$
